# Semi-Quantitative Characterization of Post-Transplant Lymphoproliferative Disorder Morphological Subtypes with [^18^F]FDG PET/CT

**DOI:** 10.3390/jcm10020361

**Published:** 2021-01-19

**Authors:** Filipe Montes de Jesus, Vibeke Vergote, Walter Noordzij, Daan Dierickx, Rudi Dierckx, Arjan Diepstra, Thomas Tousseyn, Olivier Gheysens, Thomas Kwee, Christophe Deroose, Andor Glaudemans

**Affiliations:** 1 Department of Nuclear Medicine and Molecular Imaging, University of Groningen, University Medical Center Groningen, 9700 RB Groningen, The Netherlands; w.noordzij@umcg.nl (W.N.); r.a.dierckx@umcg.nl (R.D.); a.w.j.m.glaudemans@umcg.nl (A.G.); 2 Department of Hematology, University Hospitals Leuven, 3000 Leuven, Belgium; vibeke.vergote@uzleuven.be (V.V.); daan.dierickx@uzleuven.be (D.D.); 3 Department of Pathology and Medical Biology, University of Groningen, University Medical Center Groningen, 9700 RB Groningen, The Netherlands; a.diepstra@umcg.nl; 4 Department of Pathology University Hospitals Leuven, 3000 Leuven, Belgium; thomas.tousseyn@uzleuven.be; 5 Department of Nuclear Medicine, Cliniques Universitaires Saint-Luc, 1200 Brussels, Belgium; olivier.gheysens@uclouvain.be; 6 Department of Radiology, University of Groningen, University Medical Center Groningen, 9700 RB Groningen, The Netherlands; t.c.kwee@umcg.nl; 7 Department of Nuclear Medicine, University Hospitals Leuven, 3000 Leuven, Belgium; christophe.deroose@uzleuven.be

**Keywords:** post-transplant lymphoproliferative disorder, 2-[^18^F]fluoro-2-deoxy-D-glucose positron emission tomography, FDG-PET/CT, semi-quantification, standardized uptake value

## Abstract

**Background:** Post-transplant lymphoproliferative disorder (PTLD) is a complication of organ transplantation classified according to the WHO as nondestructive, polymorphic, monomorphic, and classic Hodgkin Lymphoma subtypes. In this retrospective study, we investigated the potential of semi-quantitative 2-[^18^F]fluoro-2-deoxy-D-glucose ([^18^F]FDG) PET/computed tomography (CT)-based parameters to differentiate between the PTLD morphological subtypes. **Methods:** 96 patients with histopathologically confirmed PTLD and baseline [^18^F]FDG PET/CT between 2009 and 2019 were included. Extracted semi-quantitative measurements included: Maximum, peak, and mean standardized uptake value (SUV_max_, SUV_peak_, and SUV_mean_). **Results:** Median SUVs were highest for monomorphic PTLD followed by polymorphic and nondestructive subtypes. The median SUV_peak_ at the biopsy site was significantly higher in monomorphic PTLD (17.8, interquartile range (IQR):16) than in polymorphic subtypes (9.8, IQR:13.4) and nondestructive (4.1, IQR:6.1) (*p* = 0.04 and *p* ≤ 0.01, respectively). An SUV_peak_ ≥ 24.8 was always indicative of a monomorphic PTLD in our dataset. Nevertheless, there was a considerable overlap in SUV across the different morphologies. **Conclusion:** The median SUV_peak_ at the biopsy site was significantly higher in monomorphic PTLD than polymorphic and nondestructive subtypes. However, due to significant SUV overlap across the different subtypes, these values may only serve as an indication of PTLD morphology, and SUV-based parameters cannot replace histopathological classification.

## 1. Introduction

Post-transplant lymphoproliferative disorder (PTLD) is a complication of hematopoietic stem cell (HSCT) and solid organ transplantation associated with high morbidity and mortality [1,2,3,4]. These lymphoid and/or plasmacytic proliferations comprise a broad morphological spectrum, ranging from Epstein–Barr virus drive hyperplasia to malignant monoclonal proliferations, histologically indistinguishable from B-cell (or less commonly T/NK-cell) lymphomas in immunocompetent patients [5]. Pathological diagnosis is currently based on the 2017 World Health Organization (WHO) classification, comprising four main categories: Nondestructive, polymorphic, monomorphic, and classic Hodgkin Lymphoma. Although histopathological evaluation is invaluable for the diagnosis and management of PTLD, there are inherent limitations associated with a biopsy. It remains an invasive procedure with possible periprocedural complications, may be difficult to perform in deep-seated lesions, and extensive necrotic areas may impair interpretation. Moreover, a single biopsy site/sample is not always suited to demonstrate the full morphological heterogeneity, as multiple PTLD morphologies can co-occur in the same lesion or within the same patient [5]. Certain PTLD morphologies seem to be associated with a more favorable clinical course and better response to initial treatment [6]. Nondestructive and polymorphic PTLD are associated with higher response rates to reduction of immunosuppression, commonly regarded as the cornerstone of first-line treatment, while monomorphic PTLD may require a more aggressive treatment regimen [6,7,8].

2-[^18^F]fluoro-2-deoxy-D-glucose positron emission tomography/computed tomography ([^18^F]FDG PET/CT) imaging plays an essential role and is considered the standard of care in the diagnostic workup and treatment response evaluation of PTLD patients [9,10]. Although some limitations have been classically associated with [^18^F]FDG PET/CT, such as low resolution, high radiation, and economical cost, the introduction of new scanners and more efficient protocols have addressed some of these concerns [11,12]. [^18^F]FDG PET/CT is superior to purely anatomical imaging in detecting additional extra-nodal lesions, with good diagnostic performance particularly in adults [10,13,14,15]. Another advantage of [^18^F]FDG PET/CT is the possibility for metabolic quantification, implemented through semi-quantitative measurements. In immunocompetent lymphoma patients, a high maximum standardized uptake value (SUV_max_) has been shown to predict aggressive B-cell lymphomas, as well as being a significant prognostic factor for progression-free survival and overall survival [16,17]. In PTLD, the clinical value of semi-quantitative metrics is not yet established, but the mean SUV_max_ has been reported to be higher in monomorphic PTLD than other subtypes [18,19]. 

Therefore, we performed a retrospective study to test the hypothesis that semi-quantitative parameters from [^18^F]FDG PET/CT can differentiate between PTLD morphological subtypes and thus may guide treatment choice.

## 2. Research Design

### 2.1. Patient Selection

All consecutive PTLD patients with [^18^F]FDG PET/CT at baseline were retrospectively identified from the electronic patient records between 2009 and 2019 at the University Medical Center Groningen (UMCG) and the University Hospitals Leuven (UZ Leuven). Systemic, histopathologically confirmed PTLD cases according to the 2017 WHO classification were included. In addition to imaging data, clinically relevant parameters (demographics, clinical transplantation data) were also collected [20]. The study was conducted in accordance with the ethical principles of the Declaration of Helsinki and with the approval of the Medical Ethics Review Board of the University Medical Center Groningen (201700855, 07-12-2017) and the Ethical Committee of the University Hospitals Leuven (S-62132, 12-04-2019).

### 2.2. [^18^F]FDG PET/CT Acquisition

[^18^F]FDG PET/CT scans were performed using a Siemens Biograph mCT (Siemens AG, Knoxville, TN, USA) at the UMCG and a Siemens Biograph 16 HiRez, Siemens Truepoint 40 (Siemens Healthcare, Erlangen, Germany) or GE Healthcare Discovery MI4 (GE Healthcare, Chicago, IL, USA) at the UZ Leuven. Patients fasted for a minimum of 6 h and had a glycemia between 3.3 to 14.5 mmol/L, before intravenous administration of 3 to 4.25 MBq [^18^F]FDG/kg body weight. Sixty minutes after tracer administration, a whole-body (vertex to mid-thigh) PET scan was acquired using a multi-bed position, with 70 to 180 s per bed position. Low-dose CT was performed for attenuation correction and anatomical mapping with 100 kV and 30 mAs. In a small subset of patients, a concomitant full-dose, contrast-enhanced CT (constant tube potential of 100–120 kV and automatic tube current modulation of mAs in the z-direction) was performed. All scans were reconstructed according to the specifications of The European Association of Nuclear Medicine Research (EARL) program [21,22]. EARL accreditation ensures consistent calibrations across devices and derives reconstruction settings for comparable SUVs by harmonization of SUV recoveries. Hence, it allows exchangeability and pooling of quantitative results across multiple centers [22,23,24].

### 2.3. [^18^F]FDG PET/CT Semi-Quantification

Semi-quantification was performed on a specialized software platform from Hermes Hybrid 3D (Hermes Medical Solutions AB, Stockholm, Sweden) by F.M.J. (nuclear medicine research fellow) blinded for all other results. Extracted semi-quantitative measurements included: SUV_max_ (highest uptake voxel within the volume of interest), SUV_peak_ (mean of all voxels in the highest uptake 1 mL sphere) and SUV_mean_ (mean of all voxel SUV values contained within the background volume of interest). SUV measurements were calculated according to the standard formula and subsequently corrected for plasma fasting glucose (SUV×fasting glucose in mmol/L)/5. Adjustment for lean body mass was not possible, as the height of a significant number of patients was not available. Lesion segmentation was performed with the “Tumor Finder” application in Hermes Hybrid 3D, according to PERCIST recommendations [25]. If the biopsied lesion fell under the PERCIST threshold, a volume of interest was placed manually directly on the lesion. Semi-quantification was performed on the biopsied lesion prior to excision. To accurately identify the location of the biopsied lesion, pathological or surgical reports within the electronic patient dossier were consulted. If a particular lymph node had been excised prior to [^18^F]FDG PET/CT, the adjacent lymph node was used for the analysis. In the case of multiple biopsies or multiple possible locations based on the pathological or surgical reports, the lesion with the highest SUV_max_ was used for analysis. All biopsies have been reviewed by a hematopathologist and classified according to the WHO 2017 classification.

### 2.4. Statistical Analysis

Baseline patient characteristics were summarized graphically and through descriptive statistics. Variables were graphically checked for normality. Normally distributed data were presented as the mean with standard deviation, while nonparametric data were with the median and interquartile range (IQR). The Kruskal–Wallis test was used to compare SUV_max_, SUV_peak_, and SUV_mean_ at the biopsy site of the different PTLD morphological subtypes. Post-hoc-adjusted statistical significance was done with Bonferroni correction. Receiver operating characteristic (ROC) analysis was performed to determine the area under the ROC curve (AUC) and optimal cut-off between other PTLD morphologies (nondestructive and polymorphic PTLD) and monomorphic using SUV_max_, SUV_peak_, and SUV_mean_. The 95% confidence interval (95%CI) was calculated for the area under the curve. Statistical significance was set at *p* ≤ 0.05. Statistical and graphical analysis were performed using SPSS, version 23.0 (IBM Corporation, Armonk, NY, USA). ROC analysis was performed with MedCalc (MedCalc Software Ltd., Belgium) using the DeLong et al. method for comparison of the ROC curves [26].

## 3. Results

### 3.1. Patient Characteristics

**Figure 1:**
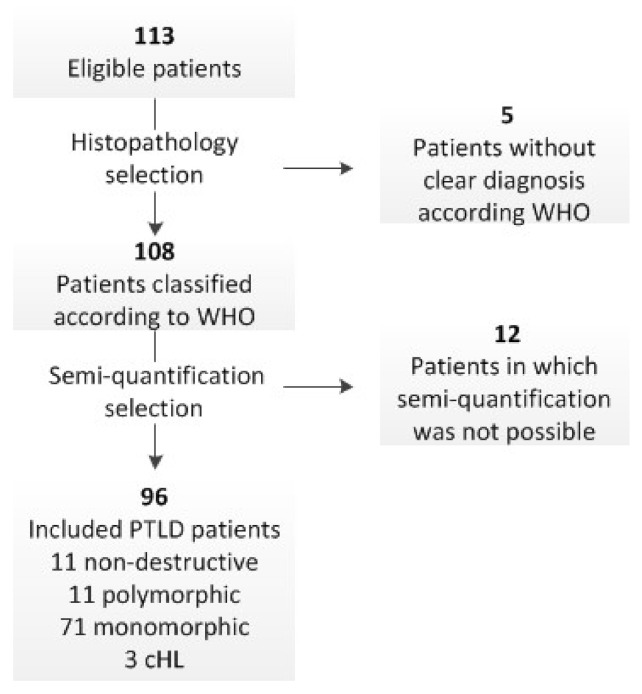
Consort diagram. WHO—World Health Organization Classification of Tumours of Haematopoietic and Lymphoid Tissues 2017.

From the electronic patient record files, 113 patients were identified (59 from UMCG and 54 the UZ Leuven). Five patients without histopathological PTLD confirmation according to the WHO 2017 classification were excluded: Three diagnoses based on cytology, one indolent lymphoma, and one unclear diagnosis due to necrosis. An additional 12 patients were excluded as [^18^F]FDG PET/CT semi-quantification was not possible: Three scans without focal FDG uptake at the biopsy site (two T-cell lymphoma and one hepatic lesion), three with complete resection of the biopsied lesion prior to [^18^F]FDG PET/CT, three with inadequate image reconstruction, two without reported fasting glucose prior to [^18^F]FDG PET/CT scan, and one in which the biopsy was performed at a surgically operated area 5 days prior to the [^18^F]FDG PET/CT. Finally, 96 patients were included in this study, including one case of relapse 6 years after the initial PTLD diagnosis (Figure 1). Sixteen were pediatric patients and 79 were adults. There were 55 males (58%) and 40 females (42%), with a median age at diagnosis of 50 years (range: 1–80). The most frequent organ transplanted was the kidney (33.6%), followed by lung (22.1%), liver (17.9%), HSCT (11.6%), heart (7.4%), and multi-organ (7.4%). Morphology was predominantly monomorphic (76%) (Table 1). Results for classic Hodgkin Lymphoma PTLD patients were included without statistical analysis as our cohort only included three cases.

### 3.2. Semi-Quantitative Measurements

**Figure 2:**
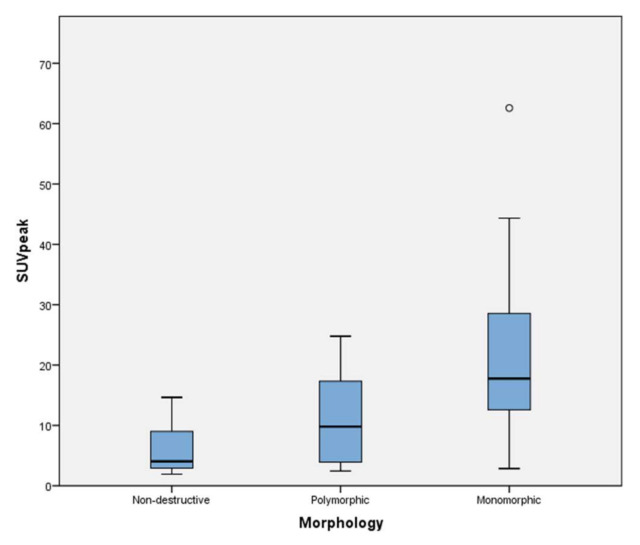
Box-plot distribution peak standard uptake value (SUV_peak_).

Overall, median SUVs were highest for monomorphic PTLD followed by polymorphic and nondestructive subtypes. SUV_max_ was highest in monomorphic PTLD (20.9, IQR:16), compared to polymorphic (13.2, IQR:15.9) and nondestructive (5.1, IQR:6.8). The same was true for SUV_peak_ (17.8, IQR:16/9.8, IQR:13.4/4.1, IQR:6.1) and SUV_mean_ (9.4, IQR:7.8/6.2, IQR:6.1/4, IQR:4.2) in monomorphic, polymorphic, and nondestructive PTLD, respectively. Finally, for classic Hodgkin Lymphoma, the median SUV_max_, SUV_peak_, and SUV_mean_ were 7.6, 6.7, and 5.3, respectively (Table 2). There was a considerable SUV overlap across the different morphologies. Particularly, minimum SUVs were similar for all subtypes (Figure 2).

After Bonferroni correction, differences in SUV_peak_ measurements retained statistical significance between nondestructive vs. monomorphic and polymorphic vs. monomorphic (*p* ≤ 0.01 and *p* = 0.04, respectively), while for SUV_max_ and SUV_mean_, only differences between nondestructive vs. monomorphic remained significant (*p* ≤ 0.01) (Table 3). However, there was a trend toward significance for polymorphic vs. monomorphic using SUV_max_ (*p* = 0.06). Median SUV differences between nondestructive and polymorphic subtypes were not statistically significant with any of the SUV parameters evaluated.

Statistical analysis (data not shown) was also performed in the subset of patients in whom direct correlation of the biopsied lesion and the SUV measurements was possible. In this analysis, 78 patients were included, 83.8% of the total cohort. The results observed were the same as with the total cohort. Therefore, only the statistical analysis of the total cohort is discussed. Additionally, SUVs derived from hottest lesion and nonglycemia-corrected SUVs were analyzed (data not shown). In both cases, a similar trend was observed to the one already presented (median SUVs from monomorphic PTLD were the highest followed by polymorphic and nondestructive subtypes, with a statistically significant difference between monomorphic and nondestructive PTLD, *p* ≤ 0.05). The only exception was with SUV_peak_ between monomorphic and polymorphic, which did not retain significance. It is noted that in 68% of the cases, the biopsied lesion was also the hottest lesion. 

### 3.3. Receiver Operating Characteristic Analysis

**Figure 3:**
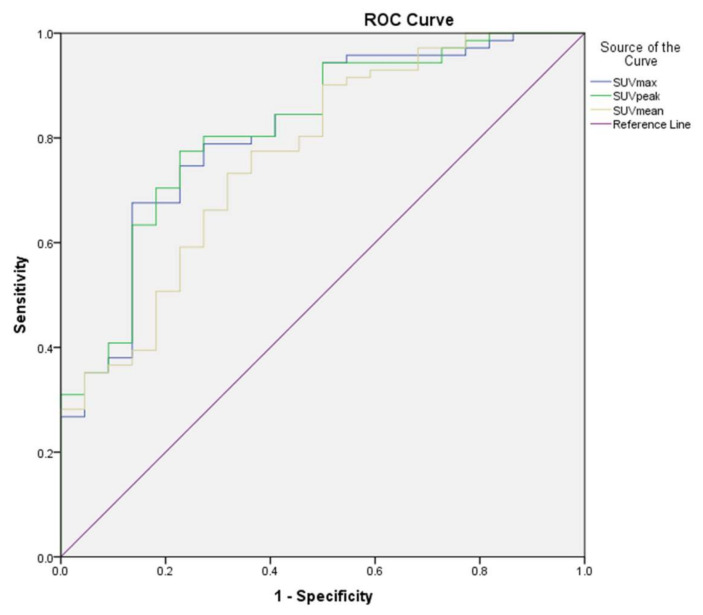
Receiver operating characteristic other-post-transplant lymphoproliferative disorder (PTLD) vs. monomorphic PTLD.

The area under the ROC curve for other-PTLD (nondestructive and polymorphic) vs. monomorphic was 0.81 (CI95%: 0.72–0.89, *p* ≤ 0.01) for SUV_max_ and 0.82 (CI95%: 0.72–0.89, *p* ≤ 0.01) for SUV_peak_ (Figure 3). For SUV_mean_, the AUC was 0.77 (CI95% 0.67–0.85, *p* ≤ 0.01). The pairwise comparison of ROC curves showed that the AUC for SUV_mean_ was significantly lower than those of SUV_max_ and SUV_peak_ (*p* = 0.03 and *p* = 0.05). Differences between AUCs from SUVmax and SUVpeak were not statistically significant (*p* = 0.51). Considering the statistically similar AUC for SUV_max_ and SUV_peak_, as well as the superior performance of SUV_peak_, to differentiate the different PTLD subtypes, only SUV_peak_ is discussed further in this paper. A SUV_peak_ cut-off of 12.2 reached the highest possible combination for sensitivity and specificity with 77% each. A SUV_peak_ above 24.8 was always indicative of a monomorphic PTLD in our study, corresponding to 25% of biopsied lesions.

## 4. Discussion

**Figure 4:**
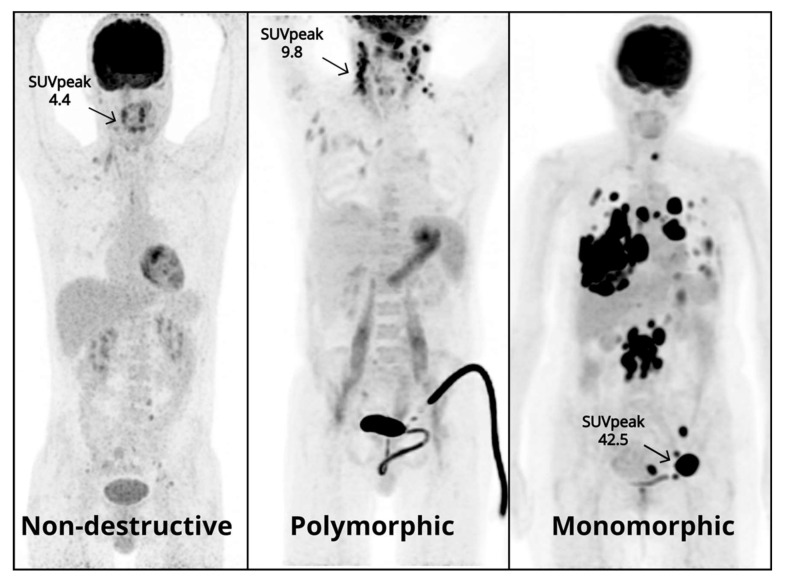
Representative PTLD patient for each morphological subtype with SUV_peak_ at biopsy site.

We investigated whether semi-quantification of the biopsied lesion could differentiate between the different PTLD morphological subtypes. SUV_peak_ at the biopsy site was significantly higher in monomorphic PTLD than in polymorphic and nondestructive subtypes (*p* = 0.04, *p* ≤ 0.01) and a SUV_peak_ greater than or equal to 24.8 is indicative of monomorphic PTLD (Figure 4). However, due to the significant overlap in SUV across the different subtypes, these values may only serve as an indication of PTLD morphology and cannot replace current histopathological classification.

Although histopathological confirmation remains necessary for PTLD diagnosis, [^18^F]FDG PET/CT may aid lesion selection or potentially be used to guide biopsies. Metabolic as well as morphological characterization of the target lesion is of particular importance in PTLD patients, as multiple morphological subtypes may be present in the same or in different lesions [5]. If we consider that first-line treatment of nondestructive PTLD may be limited to reduction of immunosuppression while monomorphic PTLD usually requires a more aggressive treatment regimen, it is essential to select the most representative lesion for biopsy [27]. Based on our cohort, an SUV_peak_ greater than 24.8 appears be indicative of monomorphic PTLD, and biopsy should be preferentially performed in lesions with higher SUVs. The value of [^18^F]FDG PET/CT-guided biopsy with an automated robotic biopsy arm has been evaluated by Radhakrishnan and collegues, where it has been reported as a feasible technique to accurately target metabolically active lesions. Furthermore, preliminary results from an ongoing trial in lymphoma patients indicates that [^18^F]FDG PET/CT-guided biopsy is a potential tool for timely and accurate diagnosis [28,29].

Previous studies have suggested that a higher SUV_max_ in monomorphic PTLD represents the more aggressive nature of this subtype [18,19]. Although we refrain from a direct correlation between a higher SUV and a more aggressive subtype, it is nevertheless interesting to observe an increasing metabolic activity pattern across the different PTLD subtypes, in which: Monomorphic > polymorphic > nondestructive. A molecular basis may explain this pattern. Although genomic and phenotypic analysis of PTLD is limited, monomorphic PTLD has been reported to display a higher number of mutations compared to polymorphic and nondestructive subtypes [30,31]. In a study by Morscio et al., chromosomal abnormalities were reported in 72% of monomorphic PTLD cases, 15% in polymorphic PTLD, and none in nondestructive PTLD cases [31]. In particular, TP53 mutations, which have been associated with increased FDG-uptake, are more frequently reported in monomorphic PTLD [30,32]. Other genetic abnormalities in monomorphic PTLD, which may account for the increase in SUV, include the increased BCL2/MYC expression and BCL2/BCL6/MYC rearrangements [33]. Therefore, differences in mutation landscape as well as microenvironment may be one of the drivers responsible for the differences in glucose metabolism across PTLD subtypes.

Previous studies seem to support our current findings. In a study with 27 biopsy-confirmed PTLD patients, Takehana et al. reported that monomorphic/classic Hodgkin Lymphoma PTLD subtypes had a significantly higher mean SUV_max_ compared to polymorphic PTLD [18]. Similarly, in a pediatric PTLD population study including 34 patients, mean SUV_max_ was higher in monomorphic PTLD than in nondestructive and polymorphic subtypes, yet this difference was not statistically significant [19]. Both groups concluded that the higher mean SUV_max_ in monomorphic PTLD was suggestive of a more aggressive nature of this subtype. Nevertheless, our study differs fundamentally from the previous studies in several aspects. We have included 93 patients from two academic hospitals, and unlike previous studies, our primary outcome was focused on the characterization of the different PTLD morphologies using semi-quantitative measurements. We have also extracted the SUV measurements from the diagnostic biopsy site, rather than calculating the mean of all FDG-avid lesions. Considering the pathological heterogeneity of PTLD and the limitations of [^18^F]FDG PET/CT in differentiating tumor lesions from infection or inflammation, choosing the biopsied lesion for semi-quantification allows direct correlation between histological subtype and semi-quantitative [^18^F]FDG PET/CT parameters. Finally, our analysis was performed on reconstructed images according to EARL recommendations, allowing for comparison and validation with future cohorts.

The retrospective nature of this study constitutes an inherent limitation. Despite using histopathological confirmation as a reference standard, distinction between the different morphological subtypes remains challenging and possible variations in the final diagnosis amongst pathologists cannot be ruled out, as a central review was not performed [31,34]. Due to the limited number of nondestructive, polymorphic, and classic Hodgkin Lymphoma PTLD cases, our analysis did not include a validation cohort. Additionally, when interpreting the cut-off obtained from the ROC analysis, clinicians must be aware of [^18^F]FDG PET/CT-inherent limitations as high SUV uptake may also indicate inflammation or another malignancy. 

Altogether, whole-body lesion semi-quantification with [^18^F]FDG PET/CT at diagnosis shows clinical potential but should be prospectively validated. Moreover, further research is needed to investigate the underlying mechanisms of the different metabolic patterns across PTLD morphologies. Finally, the value of semi-quantitative measures in the prognostication of PTLD patients should be investigated in analogy to immunocompetent patients. In immunocompetent lymphoma patients, high metabolic tumor volume and total lesion glycolysis have been associated with worse survival, but these measurements have not yet been evaluated in PTLD patients [35].

## 5. Conclusions

In a large PTLD patient cohort from two academic hospitals, the median SUV_peak_ at the biopsy site was significantly higher in monomorphic than in polymorphic and nondestructive PTLD subtypes. However, due to the significant overlap in SUV across the different subtypes, this metric cannot replace current histopathological classification. An SUV_peak_ ≥ 24.8 is strongly suggestive of a monomorphic PTLD subtype.

## Figures and Tables

**Table 1:** Patient characteristics (*n* = 96) *.

Age at Diagnosis (Years)	
Median	50
Range	1–80
IQR	39
**Gender**	
Male	55 (58%)
Female	40 (42%)
**Transplanted organ**	
Kidney	32 (33.6%)
Lung	21 (22.1%)
Liver	17 (17.9%)
HSCT	11 (11.6%)
Heart	7 (7.4%)
Multi-organ	7 (7.4%)
**Histology**	
Nondestructive	11 (11.5%)
Polymorphic	11 (11.5%)
**Monomorphic**	71 (76%)
Diffuse Large B-cell	50 (70.4%)
Plasmacytoma-like	8 (11.3%)
Burkitt	7 (9.9%)
T-cell	1 (1.4%)
Other/Unclear	5 (7%)
Classic Hodgkin Lymphoma	3 (3%)

IQR—interquartile range, HSCT—hematopoietic stem cell transplantation. * 1 relapsed patient (monomorphic PTLD and Classic Hodgkin Lymphoma).

**Table 2:** Semi-quantitative measurements.

Semi-Quantification	Median	IQR	Min	Max
SUV_max_				
Nondestructive (n = 11)	5.1	6.8	2.5	17.7
Polymorphic (n = 11)	13.2	15.9	3.3	31.1
Monomorphic (n = 71)	20.9	16	3.4	69.3
Classic Hodgkin Lymphoma (n = 3)	7.6		6.2	10.2
SUV_peak_				
Nondestructive (n = 11)	4.1	6.1	1.9	14.6
Polymorphic (n = 11)	9.8	13.4	2.4	24.8
Monomorphic (n = 71)	17.8	16	2.9	62.6
Classic Hodgkin Lymphoma (n = 3)	6.7		4.2	9.4
SUV_mean_				
Nondestructive (n = 11)	4	4.2	1.3	11.3
Polymorphic (n = 11)	6.2	6.1	2.2	12.9
Monomorphic (n = 71)	9.4	7.8	2.5	22.9
Classic Hodgkin Lymphoma (n = 3)	5.3		4.3	6.9

IQR—interquartile range, Min—minimum, Max—maximum.

**Table 3:** Adjusted pairwise semi-quantitative measurements.

Semi-Quantification	*p*-Value
SUV_max_	
Nondestructive vs. Polymorphic	0.41
Nondestructive vs. Monomorphic	0.01
Polymorphic vs. Monomorphic	0.06
SUV_peak_	
Nondestructive vs. Polymorphic	0.52
Nondestructive vs. Monomorphic	0.01
Polymorphic vs. Monomorphic	0.04
SUV_mean_	
Nondestructive vs. Polymorphic	0.79
Nondestructive vs. Monomorphic	0.01
Polymorphic vs. Monomorphic	0.11

## Data Availability

The datasets used and/or analyzed during the current study are available from the corresponding author on reasonable request.
